# Whole Genomic Analysis of Human G1P[8] Rotavirus Strains From Different Age Groups in China

**DOI:** 10.3390/v4081289

**Published:** 2012-08-16

**Authors:** Tsuzumi Shintani, Souvik Ghosh, Yuan-Hong Wang, Xuan Zhou, Dun-Jin Zhou, Nobumichi Kobayashi

**Affiliations:** 1 Department of Hygiene, Sapporo Medical University School of Medicine, Sapporo 060-8556, Japan; Email: t.shintani@sapmed.ac.jp (T.S.); nkobayas@sapmed.ac.jp (N.K.); 2 Wuhan Centers for Disease Prevention and Control, Wuhan 430015, China; Email: wyuanh@hotmail.com (Y.-H.W.); ice2bhi@yahoo.com.cn (X.Z.); zdj@whcdc.org (D.-J.Z.)

**Keywords:** human G1P[8] rotavirus, whole genomic analysis, China

## Abstract

G1P[8] rotaviruses are an important cause of diarrhea in humans in China. To date, there are no reports on the whole genomic analysis of the Chinese G1P[8] rotaviruses. To determine the origin and overall genetic makeup of the recent Chinese G1P[8] strains, the whole genomes of three strains, RVA/Human-wt/CHN/E1911/2009/G1P[8], RVA/Human-tc/CHN/R588/2005/G1P[8] and RVA/Human-tc/CHN/Y128/2004/G1P[8], detected in an infant, a child and an adult, respectively, were analyzed. Strains E1911, R588 and Y128 exhibited a typical Wa-like genotype constellation. Except for the NSP3 gene of E1911, the whole genomes of strains E1911, R588 and Y128 were found to be more closely related to those of the recent Wa-like common human strains from different countries than those of the prototype G1P[8] strain, or other old strains. On the other hand, the NSP3 gene of E1911 was genetically distinct from those of Y128, R588, or other Wa-like common human strains, and appeared to share a common origin with those of the porcine-like human G9 strains, providing evidence for intergenotype reassortment events. Comparisons of the amino acid residues defining the VP7 and VP4 antigenic domains revealed several mismatches between these Chinese G1P[8] strains and the G1 and P[8] strains contained in the currently licensed rotavirus vaccines Rotarix^TM ^and RotaTeq^TM^.

## 1. Introduction

Group A rotavirus (RVA) (Family *Reoviridae*, genus *Rotavirus*, species *Rotavirus A*) is a major cause of severe childhood diarrhea [1]. The RVA VP4 and VP7 outer capsid proteins elicit protective immunity against rotavirus infection, forming the basis of the current RVA vaccines [1]. To date, RVAs are classified into at least 27 G and 35 P genotypes on the basis of variations in the nucleotide sequences of their VP7 and VP4 genes, respectively [2]. In humans, G1, G2, G3, G4 or G9 strains in combination with P[4], P[6] or P[8] have been widely reported, whilst, G12 has been emerging as the sixth globally important human VP7 genotype [3,4]. Among the common human RVAs, G1P[8] strains constitute the majority of human RVA infections worldwide [3,4]. Therefore, the VP7 of G1 and VP4 of P[8] have been included in both the currently licensed oral RVA vaccines, Rotarix^TM^ (live-attenuated monovalent RVA vaccine, GlaxoSmithKline Biologicals, Belgium) and RotaTeq^TM^ (live-attenuated pentavalent RVA vaccine, Merck and Co., USA) [5].

In China, RVAs have been associated with 12.10 million cases of childhood diarrhea annually, imposing a tough burden on the national economy [6]. The most frequent RVA strains were G3P[8] and G1P[8], though other common (G2P[4], G4P[8] and G9P[8]) and uncommon (G1P[4], G1P[6], G3P[4] and G4P[6]) human strains have been also reported [7,8,9]. The Chinese government has yet to introduce routine RVA vaccination into the national childhood immunization program [6]. However, a live oral RVA vaccine, the Lanzhou lamb rotavirus (LLR) vaccine, has been licensed for use in China since 2000 [10]. To date, the currently licensed RVA vaccines, Rotarix^TM^ and RotaTeq^TM^, remain to be introduced in China.

Whole genomic analyses of common human RVA strains from different countries are essential to obtain conclusive data on their overall genetic makeup and evolution patterns [11,12]. Although G1P[8] is a predominant global genotype [3,4], the whole genomes of only a few recent human G1P[8] RVA strains from Bangladesh, India and USA have been analyzed so far [13,14,15,16]. Based on limited whole genome-based studies on common human RVAs, it has been hypothesized that a stable Wa-like genetic backbone might be circulating in majority of the recent Wa-like common human RVAs, such as G1P[8], facilitating the propagation of these strains worldwide [11,13,15]. However, whole genomic analyses of common human Wa-like RVAs from different countries across the globe are required to corroborate this hypothesis. In China, G1P[8] RVAs have been identified as one of the major causes of childhood diarrhea [7,8,9]. However, to date, there are no reports on the whole genomic analysis of the G1P[8] RVA strains from China. Therefore, to gain insights into the overall genetic makeup and evolution of the recent Chinese G1P[8] strains and compare their genetic backbones with those of common human Wa-like RVAs from other countries, the whole genomes of three human G1P[8] RVA strains, RVA/Human-wt/CHN/E1911/2009/G1P[8], RVA/Human-tc/CHN/R588/2005/G1P[8] and RVA/Human-tc/CHN/Y128/2004/G1P[8], detected in the city of Wuhan, central China, were analyzed in the present study. 

In the present study, the three Chinese G1P[8] strains were selected among RVAs from infants, children and adults, respectively. Despite generally affecting infants and children, common human RVAs, such as G1, G2 and G3 strains, have also been associated with diarrhea in adults [8,9,17,18]. Limited studies, based on analyses of the VP4 and VP7 genes, have demonstrated a close genetic relationship between RVAs from children and adults [8,18], but the whole genomes of human RVAs from adults and children have never been compared before.

## 2. Results, Discussion and Conclusion

**Figure 1:**
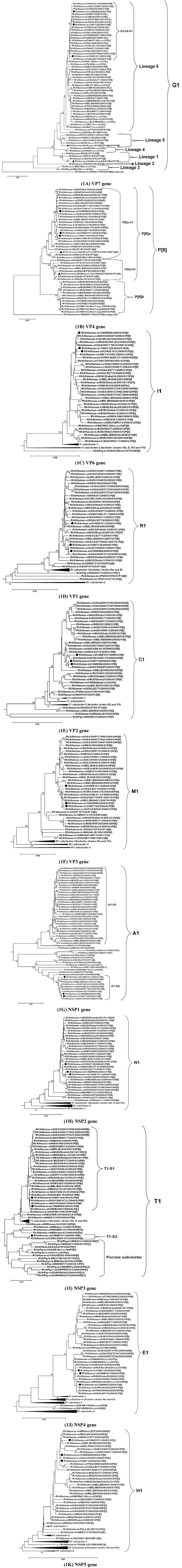
(**A-K**) Phylogenetic trees constructed from the nucleotide sequences of VP7, VP4, VP6, VP1-3 and NSP1-5 genes of rotavirus strains RVA/Human-wt/CHN/E1911/2009/G1P[8], RVA/Human-tc/CHN/R588/2005/G1P[8],RVA/Human-tc/CHN/Y128/2004/G1P[8], with those of the other group A rotavirus strains. Although strains representing all the RV-A genotypes were included in the phylogenetic analyses to prepare the dendograms, only those relevant to the present analysis are shown in Figure 1A-K. In all trees, positions of strains E1911, R588 and Y128 are shown by closed circles. In Figure 1A and 1B, triangles indicate the G1 and P[8] RVA strains contained in the currently licensed rotavirus vaccines Rotarix™ and RotaTeq™. Scale bar, 0.05 substitutions per nucleotide. Bootstrap values less than 85% are not shown.

By nucleotide sequence identities and phylogenetic analyses of the nearly full-length nucleotide sequences, the VP7-VP4-VP6-VP1-VP2-VP3-NSP1-NSP2-NSP3-NSP4- NSP5 genes of strains E1911, R588 and Y128 were assigned to the G1-P[8]-I1-R1-C1-M1-A1-N1-T1-E1-H1 genotypes, respectively (Figure 1A–K). Therefore, all the three Chinese G1P[8] RVA strains exhibited a typical Wa-like genotype constellation. With the exception of the NSP3 gene of strain E1911, strains E1911, R588 and Y128, detected in an infant, a child and an adult, respectively, were found to be closely related (nucleotide sequence identities of 97.5–99.8%) to each other (Figure 1A–K). 

The VP7 genes of strains E1911, R588 and Y128 exhibited high nucleotide sequence identities (99%) to those of several other recent G1 strains. Phylogenetically, the VP7 genes of the Chinese G1P[8] strains clustered into a subcluster (shown as G1-L6-S1) that primarily consisted of G1 strains from China and Japan within G1-Lineage 6, different from those of the G1 strains contained in the RVA vaccines Rotarix^TM^ (Lineage 5) and RotaTeq^TM^ (Lineage 1) (Figure 1A). By multiple alignment, the deduced amino acid sequences of the VP7 of the Chinese G1P[8] strains exhibited 17–18, 14–15 and 21–22 mismatches with those of the prototype G1P[8] strain Wa, VP7 of Rotarix^TM^ and G1 component of RotaTeq^TM^, respectively. To date, the RVA P[8] strains have been classified into two genetically distinct subtypes, P[8]a and P[8]b (also referred to as OP354-like RVAs) [19]. The VP4 genes of strains E1911, R588 and Y128 were closely related (nucleotide sequence identities of 99%) to those of the recent P[8]a strains from different countries, and phylogenetically, appeared to cluster within the same subcluster (shown as P[8]a-S1) as the P[8] component of RotaTeq^TM^, whilst the VP4 gene of Rotarix^TM^ was found to cluster into the other subcluster (designated as P[8]a-S2) within the P[8]a lineage (Figure 1B). 

**Figure 2:**
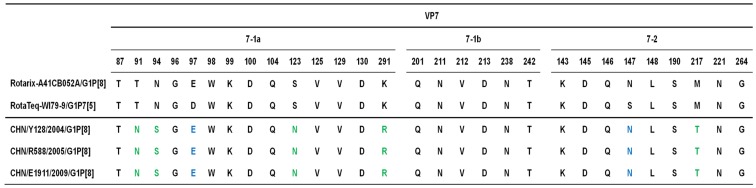
Alignment of the amino acid residues defining the neutralization domains (designated as 7-1a, 7-1b and 7-2 [24]) of VP7 between the G1 strains in Rotarix™ and RotaTeq™ and Chinese RVA strains Y128, R588 and E1911. Green indicates the residues that differ from those of both Rotarix™ and RotaTeq™. Blue indicates the residues identical to those of Rotarix™, but different from those of RotaTeq™.

**Figure 3:**
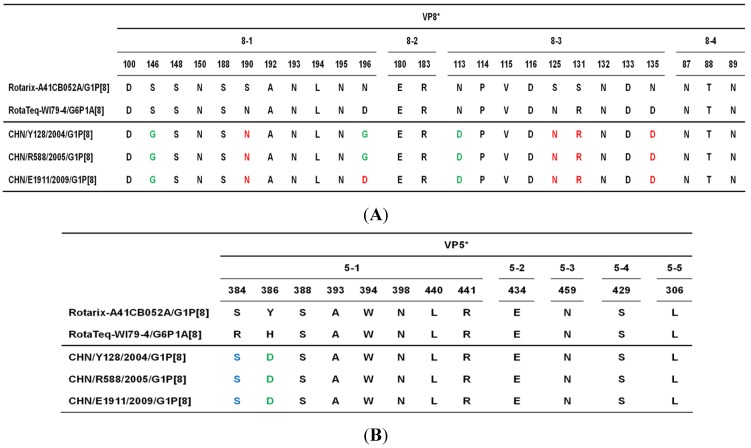
Alignment of the amino acid residues corresponding to those defining the VP4 neutralization domains (designated as 8-1, 8-2, 8-3 and 8-4 in the VP8* subunit (**A**) and 5-1, 5-2, 5-3, 5-4 and 5-5 in the VP5* subunit (**B**) of VP4) [24]) between the P[8] strains in Rotarix™ and RotaTeq™ and Chinese RVA strains Y128, R588 and E1911. Green indicates the residues that differ from those of both Rotarix™ and RotaTeq™. Red indicates the residues identical to those of RotaTeq™, but different from those of Rotarix™. Blue indicates the residues identical to those of Rotarix™, but different from those of RotaTeq™.

Deduced amino acid residues defining the RVA VP4 and VP7 epitopes have been identified by neutralization escape mutants and identifying surface exposed amino acid residues that show intergenotypic variability among prevalent human G- and P- genotypes [20,21,22,23,24]. With the exception of a single residue in VP4 of E1911, these amino acid residues were conserved among the three Chinese G1P[8] strains (Figure 2, Figure 3). Recently, the nucleotide sequences of the VP4 and VP7 genes of the G1 and P[8] strains contained in the currently licensed rotavirus vaccines Rotarix^TM^ and RotaTeq^TM^ have been reported [24,25], allowing us to compare for the first time these genes with those of the Chinese G1P[8] strains. With the amino acids defining the VP7 epitopes of G1 strains in Rotarix^TM^ and RotaTeq^TM^, the Chinese G1P[8] strains differed in 5 and 7 residues, respectively (Figure 2). Alignment of the amino acid residues defining the VP4 neutralization domains revealed 8 mismatches between the Chinese G1P[8] strains and the P[8] strain in Rotarix^TM^ (Figure 3). On the other hand, strains Y128 and R588 differed in 5 residues, whilst strain E1911 exhibited 4 mismatches with those in the VP4 of the P[8] strain in RotaTeq^TM^ (Figure 3).

Among the other genes, the VP1-3, VP6, NSP2 and NSP4-5 genes of strains E1911, R588 and Y128 were closely related (nucleotide sequence identities of 97–99%) to those of several Wa-like common human RVA strains, such as G1, G3, G9, and/or G12, detected in the 2000s from different countries (Figure 1C-F, H and J-K). Phylogenetically, the NSP1 genes of the recent Wa-like common human RVAs appeared to be grouped into two distinct subclusters, designated as A1-S1 and A1-S2, within genotype A1 (Figure 1G). The NSP1 genes of strains E1911, R588 and Y128 were found to be closely related (nucleotide sequence identities of 97-99%) to those of the recent G1P[8] and G3P[8] strains within subcluster A1-S2 (Figure 1G). The NSP3 genes of strains Y128 and R588 shared high nucleotide sequence identities (97-99%), and phylogenetically, clustered with several recent Wa-like common human G1,G3, G9 and G12 strains to form a subcluster (shown as T1-S1) (Figure 1I). On the other hand, the NSP3 gene of strain E1911 shared low nucleotide sequence identities of 89.2% with those of Y128 and R588, and exhibited a maximum nucleotide sequence identity of 99.5% to that of G9P[8] strain RVA/Human-tc/KOR/CAU09-376/2009/G9P[8] from South Korea. Phylogenetically, the NSP3 genes of strains E1911 and CAU09-376 clustered together (shown as subcluster T1-S2), near the subcluster of porcine-like human G9 strains from India (strains RVA/Human-wt/IND/RMC321/ 1990/G9P[19], RVA/Human-wt/IND/mani-97/2006/G9P[19] and RVA/Human-wt/IND/mcs/13-07/ 2007/G9P[6]) [26,27], and were genetically distinct from those of strains Y128, R588, other recent Wa-like common human strains, and the prototype G1P[8] strain, Wa (Figure 1I). Strain E1911 was found to share nucleotide sequence identities of 93.3%, 92.4% and 91.3% with the NSP3 genes of strains mani-97, mcs/13-07 and RMC321, respectively.

Taken together, with the exception of the NSP3 gene of E1911, the genomes of the Chinese RVA G1P[8] strains E1911, R588 and Y128 were found to be more closely related to those of the recent Wa-like common human strains, such as G1, G3, G9, G12, from different countries than those of the prototype G1P[8] strain Wa, or other old Wa-like strains (Figure 1A-K). On the other hand, phylogenetically, the NSP3 gene of strain E1911 clustered near those of the porcine-like human G9 strains from India, and taken together, these strains appeared to share a more common origin with those of the porcine RVAs than those of the Wa-like common human strains (Figure 1I). Since the remaining genes of E1911 were closely related to those of Y128 and R588, it is likely that strain E1911 acquired its NSP3 gene through inter-genotype reassortment events. The infant infected with strain E1911 lived in the central city of Wuhan, and therefore, it is unlikely that he came in direct contact with a pig. It may be possible that the infant was infected from food or water contaminated with strain E1911, or from an adult who came in close contact with pigs. However, analysis of the NSP3 genes of locally circulating and other Chinese porcine RVA strains might be required to pinpoint the exact source of the NSP3 gene of E1911. To date, the NSP3 gene sequences of only a single porcine and a few human RVA strains are available from China, as evident from the GenBank database. Recently, the whole genomes of four Chinese human G3P[8] RVA strains have been sequenced in our laboratory (unpublished data). None of these human RVA strains from China were found to possess a porcine-like NSP3 gene [28,29, unpublished data]. Nevertheless, genetic analyses of several human and porcine strains might be required to determine as to whether the porcine-like NSP3 genes are common in RVAs prevailing in Chinese children, or strain E1911 is rare in nature.

In conclusion, whole genomic analyses of the recent Chinese G1P[8] strains revealed a stable Wa-like genetic backbone that might be circulating in majority of the recent Wa-like common human RVAs, such as the G1P[8], G3P[8], G4P[8] and G9P[8] strains, worldwide. It has been hypothesized that RVAs with this genetic backbone have the ability to propagate extremely well in the human host, as evidenced from the detection of large numbers of Wa-like human RVA strains across the globe [11,13,15]. Comparison of the whole genomes of the Chinese G1P[8] strains from different age groups revealed a close genetic relationship among these RVAs, suggesting that genetically identical G1P[8] strains might be circulating among children and adults in Wuhan city, China. Although the present study provided important insights into the origin and overall genetic makeup of the widely circulating human G1P[8] RVA strains in China, it was limited to only three recent strains. Whole genomic analyses of additional RVA strains from different geographical regions might be required to gain a proper understanding of the evolutionary dynamics of the primary RVA strains in China. 

The currently licensed RVA vaccines, Rotarix^TM^ and RotaTeq^TM^, have been found to be effective against the common human RVA strains, resulting in substantial declines in rotavirus and/or diarrhea-related hospitalization in many countries [30]. Although there were concerns on the efficacy of the monovalent G1P[8] vaccine, Rotarix^TM^, against completely heterotypic strains, Rotarix^TM^ was shown to be effective against the common human G2P[4] RVAs in Brazil [30,31]. However, these vaccines are yet to be introduced in China. Since the Chinese G1P[8] RVAs share close genetic similarity with those of recent G1P[8] RVAs from other countries where routine RVA vaccination has yielded good results, it might be possible that these vaccines will cause a sharp reduction in the number of cases of RVA associated diarrhea in China. However, comparisons of the amino acid residues defining the VP7 and VP4 antigenic domains revealed several mismatches between the recent Chinese G1P[8] strains and the G1 and P[8] strains contained in Rotarix^TM^ and RotaTeq^TM^. Implications of these changes on the efficacy of these vaccines, if any, need to be monitored after their introduction in China. There is evidence that genes other than VP7 and VP4 might influence the immune response in the host following RVA vaccination [3,32]. Continuous vaccine-induced immunological pressure may cause changes in these genes that are detrimental to the efficacy of the current RVA vaccines [3]. Therefore, large scale whole genome-based studies on common human RVA strains from different countries are required to identify these vaccine-induced changes in the RVA genome. To our knowledge, this is the first report on the whole genomic analysis of G1P[8] RVA strains from China.

## 3. Materials and Methods

### 3.1. Virus Strains

RVA strains RVA/Human-wt/CHN/E1911/2009/G1P[8], RVA/Human-tc/CHN/R588/2005/G1P[8] and RVA/Human-tc/CHN/Y128/2004/G1P[8] were detected in diarrheal stool samples collected from an infant, a male child and an adult in the city of Wuhan, central China, in 2009, 2005 and 2004, respectively [8,9]. Age, sex and clinical features of the patients infected with these RVA strains are shown in Table 1. Among these RVAs, strains Y128 and R588 could be successfully isolated by tissue culture in MA-104 cells. 

**Table 1:** Age, sex and clinical features of the patients infected with strains E1911, R588 and Y128.

RVA strain	Age and sex of patient	Duration of diarrhea	Clinical signs	Duration of Hospitalization
E1911	8-month-old male infant	1 day	Passing liquid stools three times a day. Mild dehydration. No vomiting or fever.	None. Treated at outpatient department.
R588	3-year-old male child	2 days	Passing liquid stools five times a day. Mild dehydration. No vomiting or fever.	None. Treated at outpatient department.
Y128	66-year-old man	1 day	Passing liquid stools seven times a day. Vomiting three times a day. Severe dehydration. No fever.	None. Treated at outpatient department.

### 3.2. RT-PCR and Nucleotide Sequencing

For RT-PCR, viral RNA was extracted from the tissue culture fluid (strains R588 and Y128) or fecal sample (strain E1911) using the QIAamp Viral RNA Mini kit (Qiagen Sciences, MD, USA). Primers used for the amplification of the VP1-4, VP6-7 and NSP2-5 genes of strains E1911, R588 and Y128 have been described previously [29,33,34]. Primers P[8]a-NSP1-11f (5'-ATG AAA AGT CTT GTG GAA GCC-3', nucleotide positions 11–31) and P[8]a-NSP1-1541r (5'-CTA CTC TAG TGC AGG GAG TC-3', nucleotide positions 1541–1522), designed from gene segment 5 of strain RVA/Human-tc/BGD/ MMC71/2005/G1P[8], were used to amplify the NSP1 genes of the Chinese G1P[8] strains. Nucleotide sequences were obtained using the BigDye Terminator v3.1 Cycle Sequencing kit (Applied Biosystems, Foster City, CA) on an automated DNA sequencer (ABI PRISM 3100). 

### 3.3. Sequence Analyses

Nucleotide sequence identities were determined as described previously [34]. Phylogenetic trees were constructed by the Neighbor-Joining method [35] using MEGA (v5.01) software. The trees were statistically supported by bootstrapping with 1000 replicates, and phylogenetic distances were measured by the Kimura two-parameter model. Multiple alignments were performed using the CLUSTAL W program [36] with default parameters. 

### 3.4. Nucleotide Sequence Accession Numbers

The GenBank accession numbers for the nucleotide sequences of the VP1-4, VP6-7 and NSP1-5 genes of strains Y128, R588 and E1911 are JQ087423-JQ087455, respectively. 

## Supplementary Files

Supplementary File 1: PDF-Document (PDF, 120 KB)
