# Supplementary material for: Whole Genomic Analysis of Human G1P[8] Rotavirus Strains From Different Age Groups in China
Source: Viruses. 2012 Aug 16;4(8):1289–304. doi: 10.3390/v4081289 (PMC3446763; doi:10.3390/v4081289)
Supplement: Supplementary File 1: — PDF-Document (PDF, 120 KB) [file viruses-04-01289-s001.pdf]

Supplementary Fig. S1. Comparison of the deduced amino acid sequences of the VP7 genes of rotavirus strains Y128, R588 and E1911 with those of selected old and recent G1 rotavirus strains from China and other countries and the G1 component of rotavirus vaccines RotaTeq™ and Rotarix™. The variable regions (designated as V1 to V9) are shaded in grey. Variable regions V5, V7, V8, and V9 correspond to the putative antigenic regions A, B, C, and F, respectively.

Country/Strain/Year/G-P combination

```
                              1             V1          V2               V3    50
CHN/Y128/2004/G1P[8]          MYGIEYTTILIFLISIILLNYILKSVTRIMDYIIYRFLLISIALFALTKA
CHN/R588/2005/G1P[8]          ..................................................
CHN/E1911/2009/G1P[8]         .....................M...................V........
USA/Wa/1974/G1P1A[8]          ........................................TV......R.
JPN/K8/1977/G1P[9]            ........................................T.......R.
JPN/KU/1978/G1P1A[8]          ........................................TV......R.
CHN/98'B43/1998/G1P[x]        .........................................V........
CHN/Chi-78/2002/G1P[x]        .........................................V........
BGD/Dhaka16/2003/G1P[8]       ............................M...........FV........
THA/CMH022/2004/G1P[8]        ...........................Q............FV........
BEL/BE00006/2005/G1P[8]       ........................................FV........
CHN/Z678/2006/G1P[8]          ..................................................
IND/61060/2006/G1P[8]         ........................................FV........
KOR/CAU219/2006/G1P[8]        .........................................V........
AUS/CK00047/2006/G1P[8]       ..................................................
USA/LB2719/2006/G1P[8]        .........................................V........
CHN/AS4/2007/G1P[8]           ..................................................
RotaTeq/G1                    ........................................TV......R.
Rotarix/G1                    ....................................S...YV......R.

                             51 V3              V4                      V5     100
CHN/Y128/2004/G1P[8]          QNYGLNIPITGSMDTVYSNSTQEGIFLTSTLCLYYPTEASNQISDGEWKD
CHN/R588/2005/G1P[8]          ..................................................
CHN/E1911/2009/G1P[8]         ........................V.........................
USA/Wa/1974/G1P1A[8]          ......L.......A..T.....EV...............T..N..D...
JPN/K8/1977/G1P[9]            ......L..........T.....EV...........N...T..N..D...
JPN/KU/1978/G1P1A[8]          ......L..........T.....EV...............T..N..D...
CHN/98'B43/1998/G1P[x]        ........................V.........................
CHN/Chi-78/2002/G1P[x]        ........................V.........................
BGD/Dhaka16/2003/G1P[8]       .....................R.EV...............T.........
THA/CMH022/2004/G1P[8]        ........................V...............T.........
BEL/BE00006/2005/G1P[8]       .....................R.EV...............T.........
CHN/Z678/2006/G1P[8]          ..................................................
IND/61060/2006/G1P[8]         ........................................T.........
KOR/CAU219/2006/G1P[8]        ......M.................V.........................
AUS/CK00047/2006/G1P[8]       .......................E..........................
USA/LB2719/2006/G1P[8]        ...................Y....V...............T.........
CHN/AS4/2007/G1P[8]           ..................................................
RotaTeq/G1                    ......L.......A..T.....EV...............T..N..D...
Rotarix/G1                    ......L..........A......................T..N......

                            101                       V6                  V7   150
CHN/Y128/2004/G1P[8]          SLSQMFLTKGWPTGSVYFKEYSNIVDFSVDPQLYCDYNLVLMKYDQNLEL
CHN/R588/2005/G1P[8]          ..................................................
CHN/E1911/2009/G1P[8]         ..................................................
USA/Wa/1974/G1P1A[8]          ..............................................S...
JPN/K8/1977/G1P[9]            ......................S.......................S...
JPN/KU/1978/G1P1A[8]          ......................S.......................S.V.
CHN/98'B43/1998/G1P[x]        ..................................................
CHN/Chi-78/2002/G1P[x]        ..................................................
BGD/Dhaka16/2003/G1P[8]       ..................................................
THA/CMH022/2004/G1P[8]        ..................................................
BEL/BE00006/2005/G1P[8]       ..................................................
CHN/Z678/2006/G1P[8]          ..................................................
IND/61060/2006/G1P[8]         ..................................................
KOR/CAU219/2006/G1P[8]        ..................................................
AUS/CK00047/2006/G1P[8]       ...............I..................................
USA/LB2719/2006/G1P[8]        ..................................................
CHN/AS4/2007/G1P[8]           ..................................................
RotaTeq/G1                    T.....................S.................F.....S...
Rotarix/G1                    ......................S...........................
```

Supplementary fig. S1 continued.

```
                                151                                               200
CHN/Y128/2004/G1P[8]            DMSELADLILNEWLCNPMDITLYYYQQSGESNKWISMGSSCTVKVCPLNT
CHN/R588/2005/G1P[8]            ..................................................
CHN/E1911/2009/G1P[8]           ..................................................
USA/Wa/1974/G1P1A[8]            ...................V..............................
JPN/K8/1977/G1P[9]              ..................................................
JPN/KU/1978/G1P1A[8]            ..................................................
CHN/98'B43/1998/G1P[x]          ..................................................
CHN/Chi-78/2002/G1P[x]          ..................................................
BGD/Dhaka16/2003/G1P[8]         ..................................................
THA/CMH022/2004/G1P[8]          ..................................................
BEL/BE00006/2005/G1P[8]         ..................................................
CHN/Z678/2006/G1P[8]            ..................................................
IND/61060/2006/G1P[8]           ..................................................
KOR/CAU219/2006/G1P[8]          ..................................................
AUS/CK00047/2006/G1P[8]         ..................................................
USA/LB2719/2006/G1P[8]          ..................................................
CHN/AS4/2007/G1P[8]             ..................................................
RotaTeq/G1                      ...................V..............................
Rotarix/G1                      ..................................................

                                201             V8                     V9         250
CHN/Y128/2004/G1P[8]            QTLGIGCQTTNVDSFETVAENEKLAIVDVVDGINHKINLTTTTCTIRNCK
CHN/R588/2005/G1P[8]            ..................................................
CHN/E1911/2009/G1P[8]           ..................................................
USA/Wa/1974/G1P1A[8]            ................MI................................
JPN/K8/1977/G1P[9]              ................M.................................
JPN/KU/1978/G1P1A[8]            ................M.................................
CHN/98'B43/1998/G1P[x]          ..................................................
CHN/Chi-78/2002/G1P[x]          ..................................................
BGD/Dhaka16/2003/G1P[8]         ........................................N.........
THA/CMH022/2004/G1P[8]          ..................................................
BEL/BE00006/2005/G1P[8]         ..................................................
CHN/Z678/2006/G1P[8]            ..................................................
IND/61060/2006/G1P[8]           ..................................................
KOR/CAU219/2006/G1P[8]          ..................................................
AUS/CK00047/2006/G1P[8]         ..................................................
USA/LB2719/2006/G1P[8]          .................I................................
CHN/AS4/2007/G1P[8]             ..................................................
RotaTeq/G1                      ................MI................................
Rotarix/G1                      .M..............M.................................

                                251                                               300
CHN/Y128/2004/G1P[8]            KLGPRENVAVIQVGGSNILDITADPTTNPQIERMMRVNWKRWWQVFYTIV
CHN/R588/2005/G1P[8]            ..................................................
CHN/E1911/2009/G1P[8]           ..................................................
USA/Wa/1974/G1P1A[8]            .................V............T.........K.........
JPN/K8/1977/G1P[9]              .................V............T.........K.........
JPN/KU/1978/G1P1A[8]            .................V............T.........K.........
CHN/98'B43/1998/G1P[x]          ..................................................
CHN/Chi-78/2002/G1P[x]          ..................................................
BGD/Dhaka16/2003/G1P[8]         ...............A..................................
THA/CMH022/2004/G1P[8]          ...............A..................................
BEL/BE00006/2005/G1P[8]         ...............A..................................
CHN/Z678/2006/G1P[8]            ..................................................
IND/61060/2006/G1P[8]           ...............A..................................
KOR/CAU219/2006/G1P[8]          ..................................................
AUS/CK00047/2006/G1P[8]         ..................................................
USA/LB2719/2006/G1P[8]          ..................................................
CHN/AS4/2007/G1P[8]             ..................................................
RotaTeq/G1                      .................V............T.........K.........
Rotarix/G1                      .................V............T.........K.........
```

Supplementary fig. S1 continued.

```
                              301                         326
CHN/Y128/2004/G1P[8]            DYINQIVQVMSKRSRSLNSAAFYYRV
CHN/R588/2005/G1P[8]            ..........................
CHN/E1911/2009/G1P[8]           ..........................
USA/Wa/1974/G1P1A[8]            ..........................
JPN/K8/1977/G1P[9]              ..........................
JPN/KU/1978/G1P1A[8]            ..........................
CHN/98'B43/1998/G1P[x]          ..........................
CHN/Chi-78/2002/G1P[x]          ..........................
BGD/Dhaka16/2003/G1P[8]         ..........................
THA/CMH022/2004/G1P[8]          ..........................
BEL/BE00006/2005/G1P[8]         ..........................
CHN/Z678/2006/G1P[8]            ..........................
IND/61060/2006/G1P[8]           ..........................
KOR/CAU219/2006/G1P[8]          ..........................
AUS/CK00047/2006/G1P[8]         ..........................
USA/LB2719/2006/G1P[8]          .......H.............I....
CHN/AS4/2007/G1P[8]             ..........................
RotaTeq/G1                      ....................S.....
Rotarix/G1                      ..........................
```
